# Schwannoma of T12 Vertebra: Case Report and Review of Literature

**DOI:** 10.1155/2000/146243

**Published:** 2000-12-22

**Authors:** P. Ramasamy, I. Shackleford, M. Al Jafari

**Affiliations:** ^1^ Warrington General Hospital, WarringtonWA5 1QG, UK

## Abstract

We report a case of schwannoma of the twelfth thoracic vertebra that presented with paraparesis. The tumour was excised, and posterior and anterior stabilisation was performed. Eighteenmonths following this procedure, the patient has solid bony union, satisfactory neurological improvement and no recurrence.


*Sarcoma* (2000) **4**, 185–190

CASE REPORT

### Schwannoma of T12 vertebra: case report and review of literature

P. RAMASAMY, I. SHACKLEFORD & M. AL JAFARI

*Warrington General Hospital, Warrington WA5 1QG, UK*

**Abstract**

We report a case of schwannoma of the twelfth thoracic vertebra that presented with paraparesis. The tumour was excised, and posterior and anterior stabilisation was performed. Eighteen months following this procedure, the patient has solid bony union, satisfactory neurological improvement and no recurrence.

**Key words:** *schwannoma, vertebra, thoracic*

### Introduction

Neurilemmomas or schwannomas are neoplasms that arise from nerve sheath cells. These rarely arise from the nerves supplying the bone. The most common bones involved are mandible, followed by sacrum. The incidence of this neoplasm in bones is reported as less than 1% of primary bone tumours.[1] We report a case of schwannoma of the twelfth thoracic (T12) vertebra in a patient who presented with paraparesis.

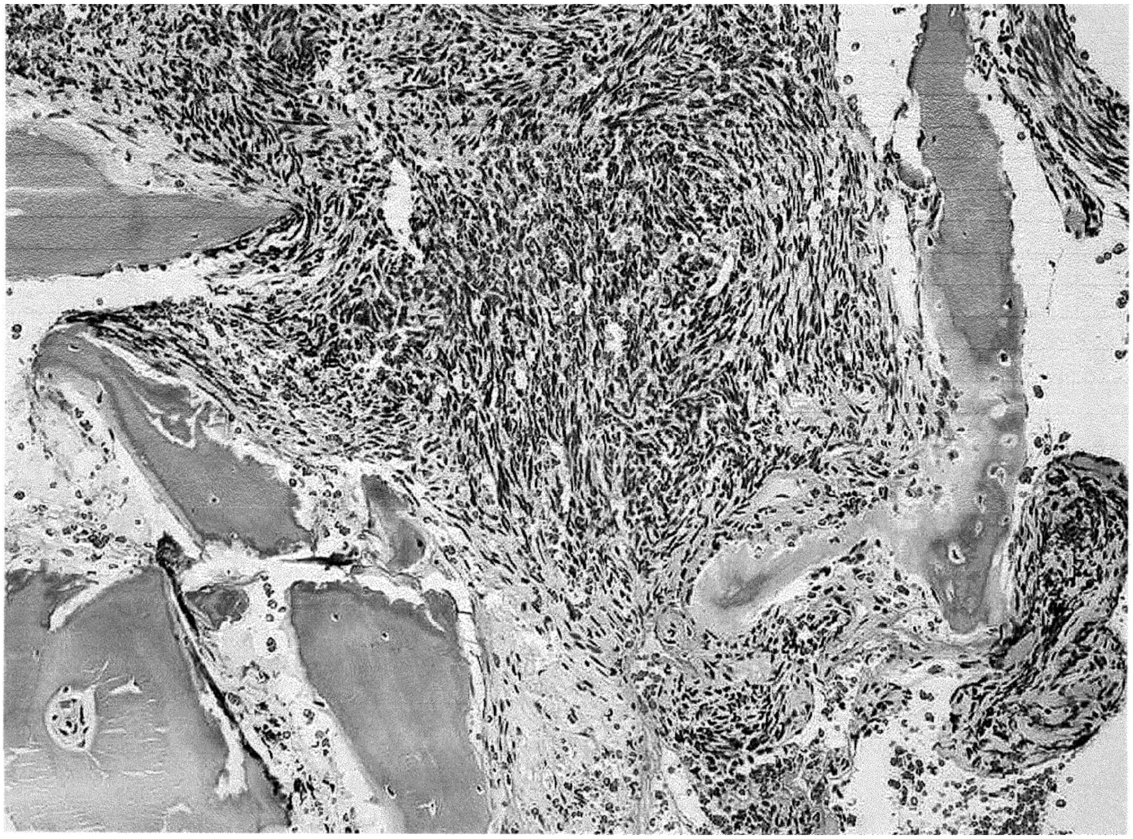

**Fig. 1.** *Photomicrograph of the schwannoma (centre) invading bone that can be seen on both sides of the lesion.*

Correspondence to: P. Ramasamy, 15 Paddock Close, Branson Court, Bradley Stoke North, Bristol BS32 0EX, UK. Tel. +44 1925 662382; Fax. +44 1925 662211; E-mail: ramassamy@hotmail.com

1357-714X print/1369-1643 online/00/040185-06 © 2000 Taylor & Francis Ltd

*DOI: 10.1080/13577140020025922*

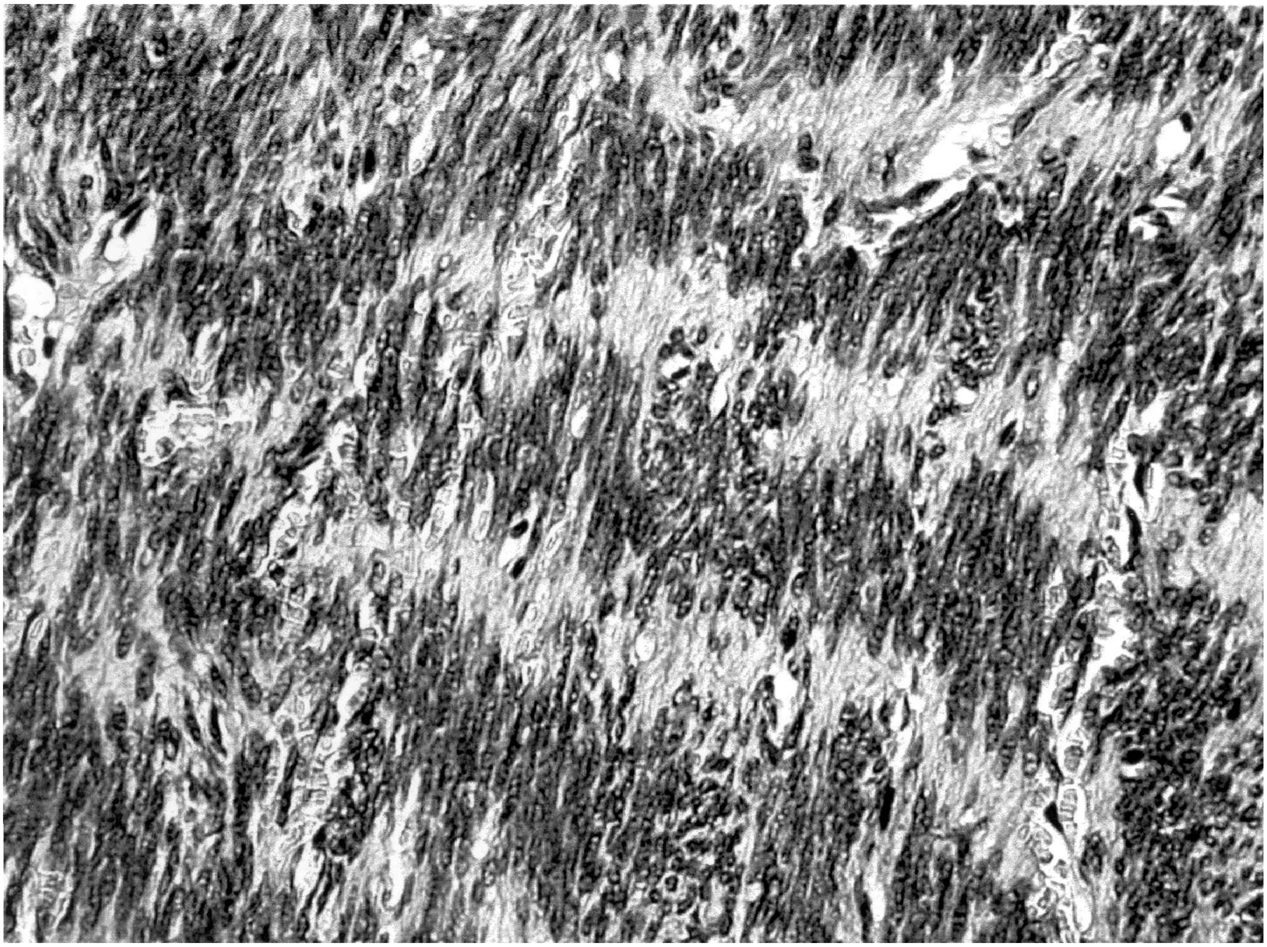

**Fig. 2.** *Higher magnification of the cellular schwannoma composed of elongated spindle cells that form a striking palisading pattern.*

### Case report

A 37-year-old man presented with back pain, weakness and numbness of both lower limbs in September 1998. He had been suffering from intermittent back pain in the past few years. In August 1998, he had developed constant back pain. His pain was mainly on his upper lumbar region and he had no leg pain. In September, he had developed weakness and numbness of his lower limbs. Weakness was worse on his right side. He did not have any bladder symptoms or perineal sensory loss.

On examination, the straight leg raising test was 50 degrees bilaterally without any leg pain. Neurological examination revealed sensory impairment of L5 and S1 dermatomes bilaterally. Motor examination revealed 0/5 power of extensor hallucis longus (EHL) and ankle evertors on the right side. On the left side, the power was 3/5 of EHL and 5/5 of evertors. There was loss of bilateral ankle jerks. There was no evidence of saddle anaesthesia and the anal tone was normal.

An urgent magnetic resonance imaging (MRI) scan was performed that showed a large soft tissue mass occupying most of the posterior part of the body of T12. The mass was extending into the spinal canal pushing the cord posteriorly. This mass had a well-defined margin all around. There was homogeneous enhancement with gadolinium.

This patient underwent excision of this lesion with anterior and posterior stabilisation in September 1998. Post-operative recovery was slow but encouraging. He had difficulty in emptying bladder in the initial post-operative period, which has since improved. Presently, he uses his abdominal muscles to facilitate bladder emptying. Power in his legs is grade 4/5 bilaterally and he walks without any aids.

Histology revealed highly cellular spindle cell tumour exhibiting marked nuclear palisading throughout the lesion without any nuclear pleomorphism. The tumour showed strong S100 staining with significant proliferation on Ki-67 staining.

### Discussion

Schwannomas arise from nerve sheath cells, particularly from sensory nerves, and are usually benign. Although it can appear in any age, the usual age group is the third or fourth decade. There appears no predilection for sex.[2] They have been reported to arise in mandible[3] (most common), sacrum,[4] lumbar,[5] thoracic[6,7] and cervical vertebrae,[8,9] femur,[10] tibia, fibula,[2] sternum,[11] calcaneum,[12] etc. The reason for the increased incidence in mandible is due to either the long intra-osseous course of the nerve or the increased predilection of schwanomas to the head

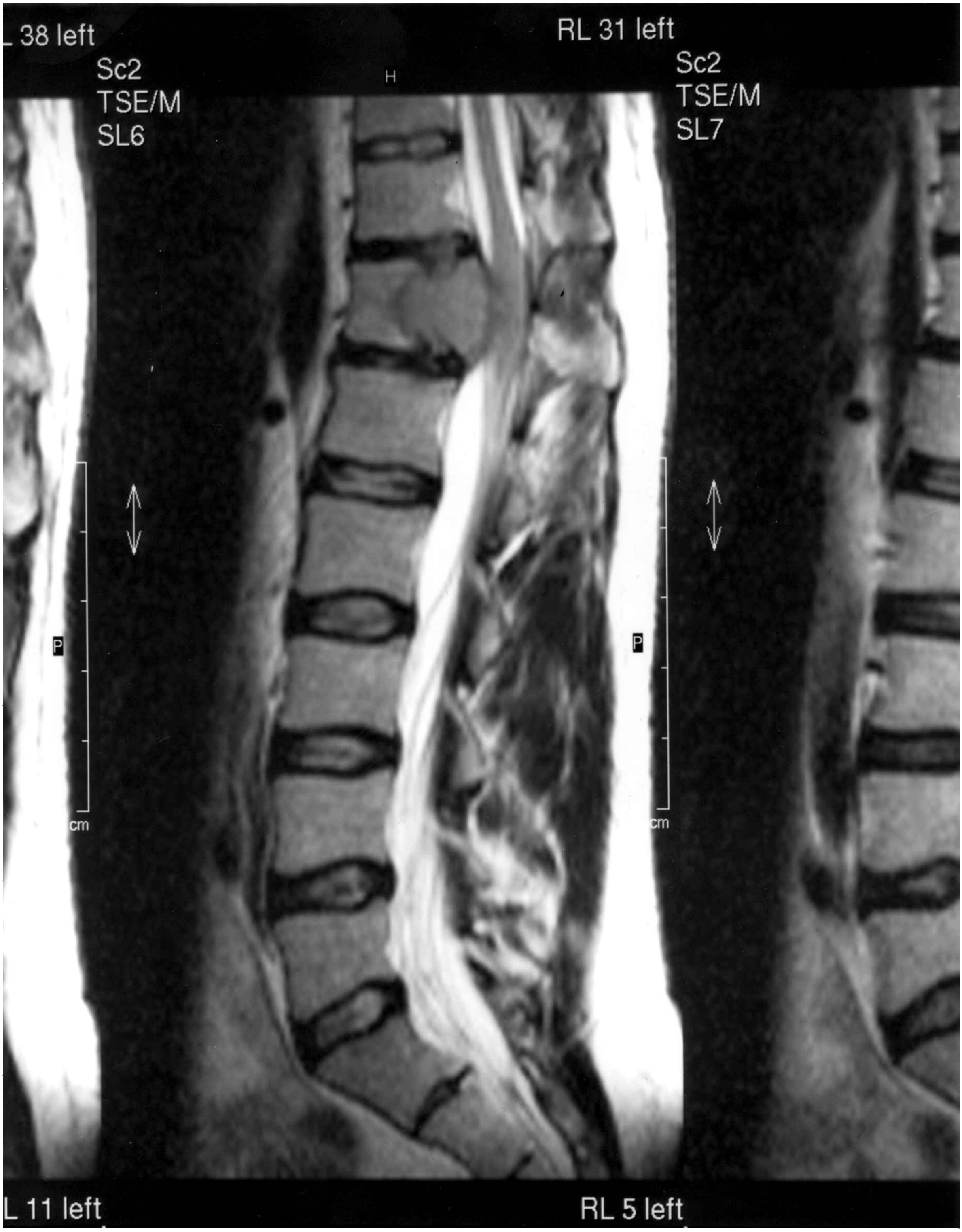

**Fig. 3.** *Saggittal T2W showing an extradural mass arising from the posterior aspect of the twelfth thoracic vertebra compressing the thecal sac (pre-operative).*

and neck region, which has a large supply of sensory nerves.[13]

*Intra-osseous schwannoma of vertebrae*

Schwannomas of the spinal column present according to the site of the vertebrae involved. The schwannoma of the cervical, thoracic and lumbar vertebrae usually present with pain[4,6,7] and/or neurological involvement.[5,8,9] The neurology differs with the level of the lesion.

Reported cases of schwannoma of cervical spine have presented with neurological involvement.[8,9] In one of the reported cases of thoracic schwannoma, the patient presented with fractured L1 vertebra following trauma but had the osteolytic lesion

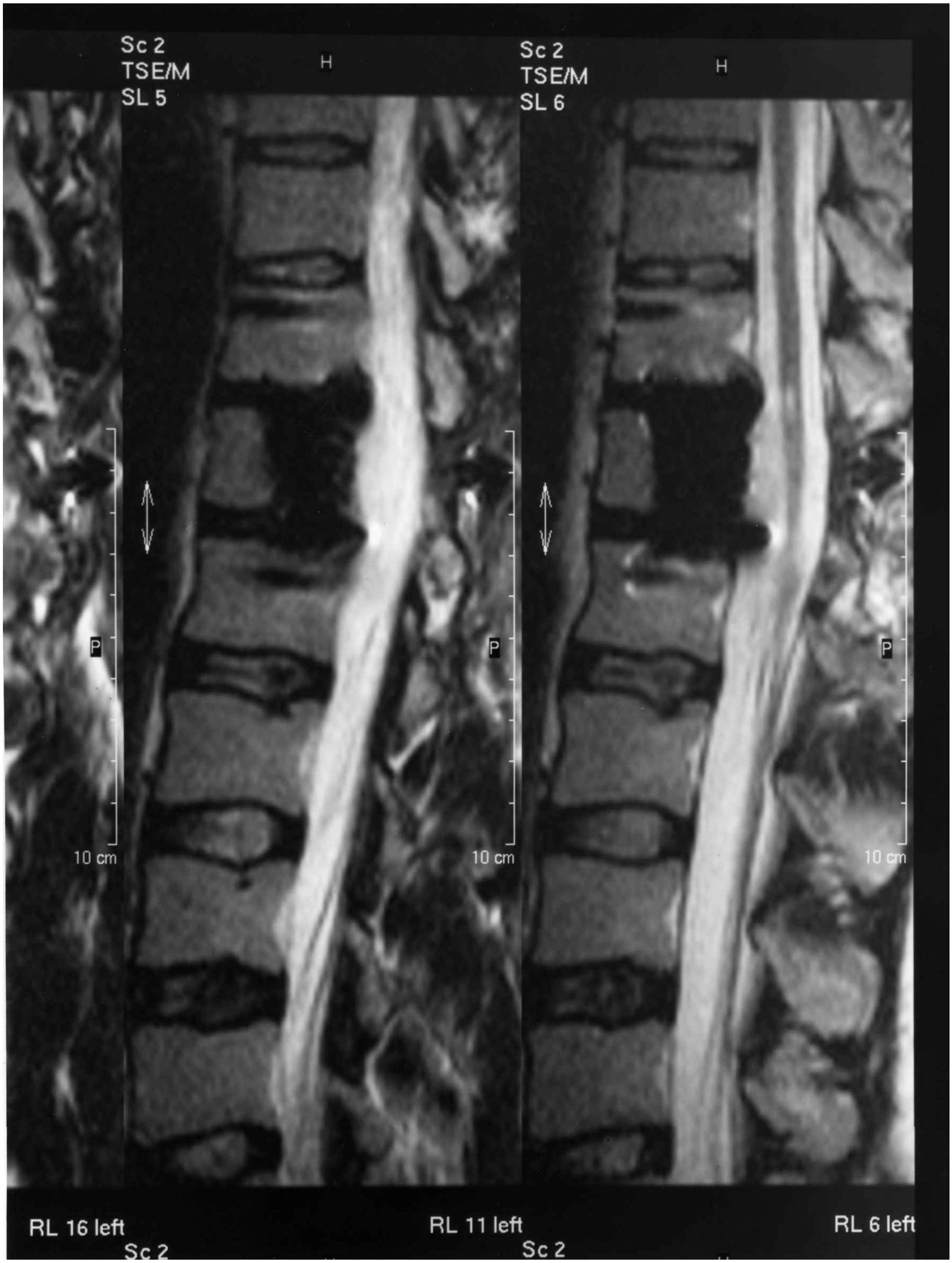

**Fig. 4.** *Saggittal T2W showing decompression of the theca (post-operative).*

(schwannoma) in T12, predisposing to the fracture.[6] In another case of thoracic schwannoma at T8 level, the patient presented with 15 years history of pain. In both cases, the neurological examination was normal. A reported case of lumbar schwannoma had neurological involvement during presentation. Schwannoma of the sacrum usually presents with low back pain, with or without sciatic pain. Cases have been reported to have presented as rectal mass during routine per rectal (P/R) examination and during barium enema examination.[1] Our patient presented with back pain and neurological symptoms as already explained.

Involvement of the bone can be explained as follows: (a) an extra-osseous tumour that can erode the bone by contiguity, (b) a tumour arising from the nutrient canal, and (c) a tumour arising centrally within the bone.[13] The differential diagnosis includes giant cell tumour, chordoma, chondrosarcoma, and metastatic deposits.

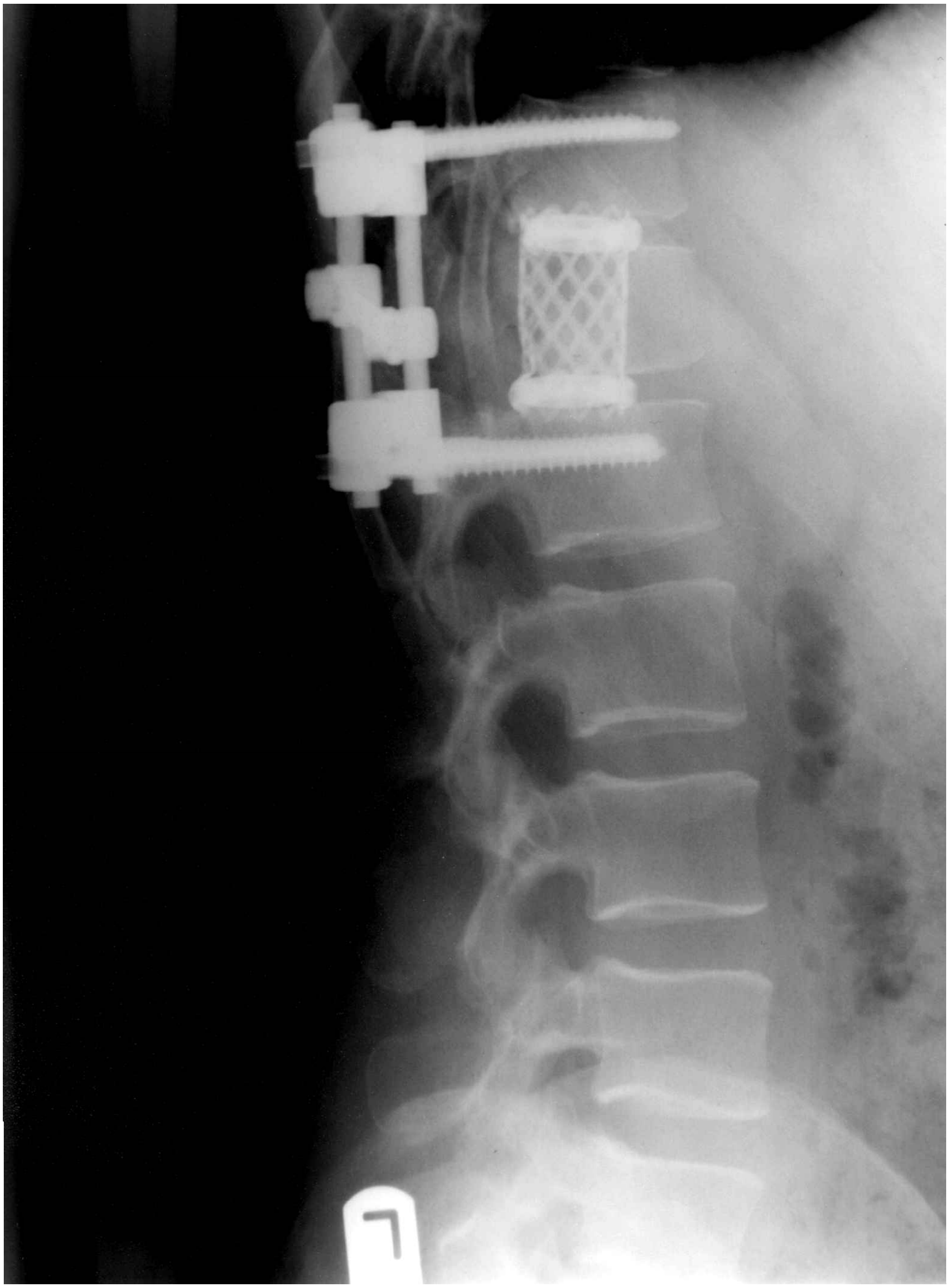

**Fig. 5.** *Post-operative X-ray showing the position of the implants.*

Recurrence is rare following surgical excision.[3,10] In our case, 1.5 years after follow-up, there is no evidence of any recurrence of the tumour. Post-operative computed tomography and MRI scans show good bony union. The clinical improvement has been stated earlier.

### References

1 Turk PS, Peters N, Libbey NP, Wanebo HJ. Diagnosis and management of giant intrasacral schwannoma. *Cancer* 1992; 70(11):2560–7.

2 Aoki J, Tanikawa H, Jujioka F, *et al.* Intraosseous neurilemmoma of the fibula. *Skeletal Radiol* 1997; 26:60–3.

3 Park YK, Kim YW, Yang MH, Kim EJ, Ryu DM. Neurilemmoma of the mandible. *Skeletal Radiol* 1999; 28:536–9.

4 Abdelwahab IF, Hermann G, Stolllman A, *et al.* Case report 564. *Skeletal Radiol* 1989; 18:466–9.

5 Chang C, Huang JS, Wang YC, Huang SH. Intraosseous schwanoma of the fourth lumbar vertebra: case report. *Neurosurgery* 1998; 43(5): 1219–22.

6 Nooraie H, Taghipour M, Arasteh MM, *et al.* Intraosseous schwannoma of T12 with burst fracture of L1. *Arch Orthopaed Trauma Surg* 1997; 116:440–2.

7 Wells F, Thomas TL, Matthewson MH, Holmes AE. Neurilemmoma of the thoracic spine. *Spine* 1982; 7(1):66–72.

8 Naidu MRC, Dinakar I, Rao KS, Ratnakar KS. Intraosseous schwannoma of the cervical spine associated with skeletal flurosis. *Clin Neurol Neurosurg* 1988; 90(3):257–9.

9 Polkey CE. Intraosseous neurilemmoma of the cervical pine causing paraparesis and treated by resection and grafting. *J Neurol Neurosurg Psychiatry* 1975; 38:776–81.

10 Sanado L, Ruiz JL, Laidler L, Polo M. Femoral intraosseous neurilemmoma. *Arch Orthopaed Trauma Surg* 1991; 110:212–3.
11 Takata K, Okuda K, Ochi M. Intraosseous neurilemmoma of the sternum. *Ann Thoracic Surg* 1999; 67:1474–6.
12 Pyati PS, Sanzone AG. Intraosseous neurilemmoma of the calcaneus. *Orthopaedics* 1996; 19(4):353–5.
13 De La Monte SM, Dorfman H, Chandra R, Malawer M. Intraosseous schwanoma: histologic features, ultrastructure and review of literature. *Hum Pathol* 1984; 15(6):551–8.
